# Diversity and Spatial Distribution of Chromophytic Phytoplankton in the Bay of Bengal Revealed by RuBisCO Genes (*rbc*L)

**DOI:** 10.3389/fmicb.2019.01501

**Published:** 2019-07-05

**Authors:** Laxman Pujari, Chao Wu, Jinjun Kan, Nan Li, Xingzhou Wang, Guicheng Zhang, Xiaomei Shang, Min Wang, Chun Zhou, Jun Sun

**Affiliations:** ^1^Research Center for Indian Ocean Ecosystem, Tianjin University of Science and Technology, Tianjin, China; ^2^Tianjin Key Laboratory of Marine Resources and Chemistry, Tianjin University of Science and Technology, Tianjin, China; ^3^Institute of Marine Science and Technology, Shandong University, Qingdao, China; ^4^Stroud Water Research Center, Avondale, PA, United States; ^5^Key Laboratory of Environment Change and Resources Use in Beibu Gulf, Nanning Normal University, Nanning, China; ^6^College of Marine Life Science, Ocean University of China, Qingdao, China; ^7^Key Laboratory of Physical Oceanography/CIMST, Ocean University of China, Qingdao, China

**Keywords:** Bay of Bengal, winter monsoon, *rbc*L gene, chromophytic phytoplankton, high throughput sequencing, morphological identification

## Abstract

Phytoplankton are the basis of primary production and play important roles in regulating energy export in marine ecosystems. Compared to other regions, chromophytic phytoplankton are considerably understudied in the Bay of Bengal (BOB). Here, we investigated community structure and spatial distribution of chromophytic phytoplankton in the BOB by using RuBisCO genes (Form ID *rbc*L). High throughput sequencing of *rbc*L genes revealed that diatoms, cyanobacteria (Cyanophyceae), Pelagophyceae, Haptophyceae, Chrysophyceae, Eustigamatophyceae, Xanthophyceae, Cryptophyceae, Dictyochophyceae, and Pinguiophyceae were the most abundant groups recovered in the BOB. Abundances and distribution of diatoms and Pelagophyceae were further verified using quantitative PCR analyses which showed the dominance of these groups near the Equator region (*p* < 0.01) where upwelling was likely the source of nutrients. Further, redundancy analysis demonstrated that temperature was an important environmental driver in structuring distributions of Cyanophyceae and dominant chromophytic phytoplankton. Morphological identification and quantification confirmed the dominance of diatoms, and also detected other cyanobacteria and dinoflagellates that were missing in our molecular characterizations. Pearson’s correlations of these morphologically identified phytoplankton with environmental gradients also indicated that nutrients and temperature were key variables shaping community structure. Combination of molecular characterization and morphological identification provided a comprehensive overview of chromophytic phytoplankton. This is the first molecular study of chromophytic phytoplankton accomplished in the BOB, and our results highlight a combination of molecular analysis targeting *rbc*L genes and microscopic detection in examining phytoplankton composition and diversity.

## Introduction

Characterization of primary productivity in oceans have been mainly focused in euphotic zones (Sigman and Hain, 2012). Marine phytoplankton are important primary producers in euphotic zones and carry out more than half of the photosynthetic carbon fixation in oceans (Falkowski et al., 1998). Therefore, it is vital to investigate the detailed composition of phytoplankton and study their spatiotemporal distributions in marine environments. Phytoplankton community structures are traditionally characterized by a suite of approaches including light microscopy (Savin et al., 2004; Vaulot et al., 2008; Joo et al., 2010), flow cytometry (Urbach and Chisholm, 1998; Collier, 2000; Cellamare et al., 2010), and pigment analysis (Bidigare and Ondrusek, 1996; Peeken, 1997). Recently, molecular methods have been applied to decipher detailed phytoplankton communities including using small subunit ribosomal RNA genes (18S rRNA) (Read et al., 2014; Needham et al., 2018). However, because of their evolutionarily conserved nature and multiple copies, rRNA genes may not provide high resolution and sometimes fail to identify organisms at the species or even at the genus level (Pochon et al., 2013; Fletcher et al., 2017). In contrast, functional genes (mostly single copy per cell) are based on selected functional aspects of organisms and likely achieve greater resolution than ribosomal RNA genes (Imhoff, 2016). Functional genes are also involved in major element metabolisms (e.g., carbon and nitrogen) and therefore provide a broad view of both genetic and functional diversity of phytoplankton, which are potentially linked to biogeochemical cycling in oceans.

Currently, functional gene markers such as *rbc*L (large subunit of RuBisCO) (Samanta and Bhadury, 2014) have been increasingly used and provided improved resolution in characterizing phytoplankton assemblages across a variety of environments. RuBisCO catalyzes the assimilation of carbon dioxide to organic carbon via the Calvin-Benson Cycle and as a fact, it has been found to be the most abundant enzyme on the Earth (Chase et al., 1993). It is principally involved in sequestering carbon dioxide from environments and reductively assimilating them into organic carbon and cellular biomass (Andersson, 1996; Yoon et al., 2000; Tabita et al., 2007). RuBisCO has four different forms (I, II, III, and IV): Form I (L8S8) contains eight large (L8) and eight small subunits (S8), and exists in all plants and some bacteria, Form II has two large units (L2) and is found in Dinoflagellates and some photosynthetic bacteria, Form III has been identified in some archaea, while form IV [also called RuBisCo-like Protein (RLP)] is widely distributed in variety of microbial groups (Kitano et al., 2001; Tabita et al., 2008). Compared to small subunits, large subunits (LSU) encoded by *rbc*L gene is widely distributed in all four forms of RuBisCO, and it contains more conserved sequences meriting it a suitable gene marker for phylogenetic identifications (Xu and Tabita, 1996). Previous studies based on *rbc*L LSU have recognized its significance in deciphering community structures of chromophytic phytoplankton and their wide distributions in different ecological niches and geographical locations in global oceans and rivers including Tampa, FL, United States and Southeastern Gulf of Mexico (Pichard et al., 1993), Mid-Atlantic Ridge and seas around Japan (Elsaied and Naganuma, 2001), California upwelling and English Channel (Bhadury and Ward, 2009), Station ALOHA in North Subtropical Gyre (Li et al., 2013), Sundarbans Mangroves reserve forest (Samanta and Bhadury, 2014), Northern South China Sea (Li et al., 2016), and Bering Sea (Endo et al., 2015). Specifically, Form I RuBisCO gene was further divided into two lineages, green and red where most of the non-green phytoplankton contain ID RubisCO and they were termed as chromophytic phytoplankton (Tabita, 1999). By using the form ID *rbc*L gene, major groups of chromophytic phytoplankton such as Bacillariophyceae, Haptophyceae, Pelagophyceae, Cryptophyceae, and Bolidiophyceae have been identified (Samanta and Bhadury, 2016). However, up to date, there is no molecular characterization available for *rbc*L genes and their distributions in the BOB.

Traditionally, the BOB is considered less biologically productive compared to its counterpart, the Arabian Sea. As the BOB is one of the largest tropical basins that is landlocked in the north, it is frequently influenced by semi-annual monsoons which create distinct seasonal changes in physicochemical parameters (Prasanna Kumar et al., 2004). As the Bay is located in a tropical region, growth of phytoplankton is usually impacted by monsoonal cloud cover and the presence of suspended sediments resulting from river discharge that may inhibit light availability for primary production (Gomes et al., 2000; Kumar et al., 2007). Additionally, vertical processes transporting nutrients into euphotic layers are generally weak in the BOB, which is likely due to strong stratification and weak upwelling (Krishna and Sastry, 1985). However, cyclones and mesoscale eddies can disturb stratification of upper layer and bring deep nutrients to the euphotic zone (Vinayachandran and Mathew, 2003; Prasanna Kumar et al., 2004; Muraleedharan et al., 2007; Reddy et al., 2008; Prasannakumar et al., 2010; Maneesha et al., 2011; Chen et al., 2013). Furthermore, BOB is characterized by a large seasonal influx of fresh water, which makes the upper water layer greatly less saline (McCreary et al., 1993; Subramanian, 1993; Kumar and Unnikrishnan, 1995; Vinayachandran and Mathew, 2003). This seasonal freshwater input is from the neighboring rivers or from excess precipitation over evaporation. A recent study showed that runoff from medium to heavy rainfall could increase phytoplankton concentration by fourfold (Maneesha et al., 2011). The BOB is also influenced by severe tropical cyclones, which occur annually during inter-monsoon periods of April–May and October–November.

The dynamic processes in the southern BOB including strong currents, intense mixing, and upwelling make BOB one of the active region of the northern Indian Ocean (Vinayachandran et al., 2004; Thushara et al., 2019). Southern BOB experiences weak salinity stratification which results in a deeper mixed layer. Moreover, monsoon circulation leads to prominent chlorophyll blooms in coastal and open ocean zone of the southern BOB. In summer, high Chl-*a* of southern BOB is linked to coastal upwelling of nutrients. The Southwest Monsoon Current (SMC) intrudes into southern BOB from Indian and Sri Lankan coasts which carries biologically rich water and supports elevated level of Chl-*a* (Vinayachandran et al., 2013). Phenomena of upwelling continue in winter monsoon at the southwestern open ocean zone of BOB which leads to the formation of moderate blooms (Vinayachandran and Mathew, 2003). However, biophysical interaction in the BOB is limited, probably due to the scarcity of observational data. Finally, BOB also experiences large seasonal freshening and semiannual reversal of the upper-layer circulation, as well as global climate events such as El Nino and Indian Ocean Dipole (IOD) (Shankar et al., 1996; Shetye et al., 1996; Kumar et al., 2007). Altogether, the BOB provides a unique environment for investigating the composition and we hypothesize that distribution of chromophytic phytoplankton and morphologically identified phytoplankton are responding to local physicochemical environmental gradients such as upwelling in southern part of the BOB with presence of different currents and freshwater influx from rivers with monsoonal precipitation in the northern BOB.

Up to date, most studies on phytoplankton in BOB have been largely restricted to satellite information derived from ocean color imagery or morphologically identified phytoplankton community composition. Although lacking detailed taxonomic information, satellite-derived phytoplankton composition provides reasonable resolution and explains the forces driving spatial distribution in BOB (Nagamani et al., 2011). Instead, microscopic approaches are able to reveal the community composition of phytoplankton with respect to spatial distribution in BOB at a given time (Boonyapiwat et al., 2008; Paul et al., 2008). Previous studies carried out in the BOB using morphologically identified phytoplankton showed diatoms as the major group which outnumbered other groups such as Chlorophyta, Pyrrophyta, Chrysophyta, and Cyanophyta (Paul et al., 2008). Further, this study also showed that morphological identified phytoplankton composition and abundance were higher in southern than in northern BOB with a higher concentration of phytoplankton present in surface water (upper 20 m depth) (Paul et al., 2008). However, identification of phytoplankton through a microscope is very challenging and some species (*Synechococcus* or *Prochlorococcus*) may escape from microscopic detection due to their pico size. Additionally, overestimation of certain phytoplankton species has been observed in previous studies (Palińska and Surosz, 2008), where conspecific individuals were misidentified as different species because of their phenotypic variations. In contrast, DNA-based molecular methods provide more feasible techniques and a large dataset of sequence repositories can be generated from environmental samples. As a result, molecular techniques including high throughput sequencing have become powerful tools for phytoplankton identification (Ebenezer et al., 2012; Xiao et al., 2014). However, very limited information on the molecular composition of phytoplankton in BOB is available, which represents a significant knowledge gap.

The main objectives of this study were: (1) to investigate community composition of chromophytic phytoplankton based on high throughput sequencing of *rbc*L genes (form ID), and (2) to understand their spatial distribution in the BOB and how they respond to environmental gradients. The high throughput sequencing analyses provided comprehensive insights into the community structure of chromophytic phytoplankton. In addition, quantitative PCR analysis of diatoms and Pelagophyceae confirmed abundances of these groups under the influence of physicochemical parameters in BOB. Finally, since *rbc*L genes form ID mainly covers algal groups including Stramenopiles, Rhodophyta and Haptophytes (Tabita et al., 2008), microscopic identification was applied to all water samples. Morphological identifications of phytoplankton provided supplementary information and revealed groups that were not detected in our molecular characterization.

## Materials and Methods

### Study Area and Sample Collection

**FIGURE 1:**
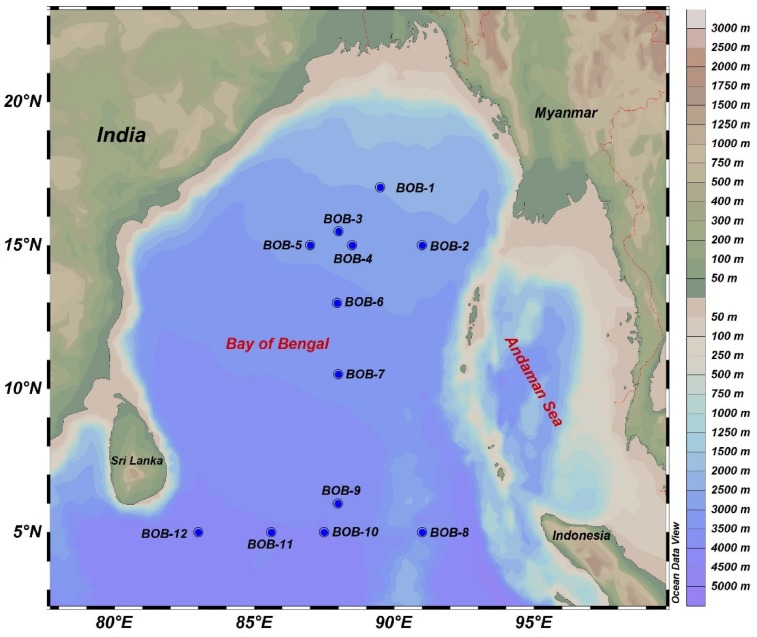
A map showing sampling sites in the BOB. Elevation/water depths are color coded with scale bar shown on the right.

Surface water samples were collected in BOB during a cruise aboard research vessel Dongfang *II* in the winter monsoon season from November 3^rd^ to December 5^th^, 2016. Surface water samples were collected from 12 selected stations (Figure 1) by a rosette multi-sampler mounted with probes and sensors measuring conductivity, temperature, and depth (Sea-Bird SBE 911Plus, Sea-Bird Electronics, United States). Subsamples (100 ml) were saved in HCL-rinsed bottles and stored at 4°C for nutrient analysis by a Technicon AA3 Auto-Analyzer (Bran+ Luebbe, Norderstedt, Germany) for phosphate (${\text{PO}}_{4}^{3-}$), ammonia (${\text{NH}}_{4}^{+}$), nitrate (${\text{NO}}_{3}^{-}$), nitrite (${\text{NO}}_{2}^{-}$), and silicate (${\text{SiO}}_{3}^{2-}$). Further, we vacuum filtered (<10 mm Hg) 500 ml of seawater for chlorophyll *a* (Chl-*a*) measurement on 25 mm GF/F filter membranes (Waterman, Florham Park, NJ, United States). After filtration, the filters were placed in aluminum foil and stored at −20°C in the dark until further analysis. Chl-*a* was extracted with 90% acetone and kept in a refrigerator at 4°C overnight, and was then analyzed using a fluorometer (CHL NA, Model # 046, Turner designs, San Jose, CA, United States). For molecular analysis, 2–3 L of seawater was filtered from all stations with 0.22-μm GTTP filter (Millipore, Eschborn, Germany) using a peristaltic pump. The filters were then flash frozen and stored in liquid nitrogen. Upon returning to the laboratory, the filters were transferred to −80°C deep freezer until DNA extraction.

For morphological identification of phytoplankton, we collected 1 L of water from each station and immediately fixed with buffered formalin (final concentration 3%). The samples were stored in a dark and cool place until further analysis. Upon returning to the laboratory, the samples were re-suspended by gentle shaking. Phytoplankton taxa were identified and quantified under a light microscope (Motic AE2000, Xiamen, China) at magnifications of 200 or 400× following the protocol described by Utermöhl (Utermöhl, 1958).

### DNA Extraction and Form ID *rbc*L Gene Amplification

Genomic DNA was extracted using DNeasyPowerWater Kits according to manufacturer’s instructions (QIAGEN, Hilden, Germany). DNA quantity and quality were checked on 1% agarose gel electrophoresis and evaluated via A260/A280 ratio using a Nanodrop 2000 Spectrophotometer (Thermo Fisher Scientific, Wilmington, DE, United States). Form ID *rbc*L gene fragments (554 bp) were amplified with previously published *rbc*L primers (Wawrik et al., 2002). PCRs were performed using a Veriti 9902 thermocycler (Applied Biosystems, Foster City, CA, United States) with each reaction containing 2 μL template DNA, 10 μL Premix Taq (Takara, Tokyo, Japan), 1 mM each primer and 6 μL of double-distilled water to make a final volume of 20 μL. PCR conditions were as follows: initial denaturation at 95°C for 5 min, 30 cycles of 95°C for 1 min, 56°C for 1 min, 72°C for 1 min, and a final extension at 72°C for 10 min. PCR reactions for each environmental sample were performed in triplicate, pooled together and subsequently purified using Universal DNA purification kits (Tiangen Biotech, Beijing, China) following the manufacturer’s instruction. All libraries were constructed and sequenced via paired-end approach on an Illumina MiSeq PE300 platform (Illumina, San Diego, CA, United States) at Allwegene Technology, Co., Ltd., Beijing, China. Subsequent image analysis, base calling and error estimation were performed using the Illumina Analysis Pipeline Version 2.6.

### qPCR Analysis

The abundance of *rbc*L genes for diatoms (Bacillariophytes) and pelagophytes were estimated using qPCR on an ABI Step One Plus Real-Time PCR system (Applied Biosystems, Foster City, CA, United States). In order to quantify Diatoms, we used primers (Forward: 5′-GATGATGARAAYATTAACTCW-3′ and Reverse: 5′-TAWGAACCTTTWACTTCWCC-3′) specified by John et al. (2007) and for Pelagophyceae, primers (Forward: 5′-CRACACWTTATTARAGACTAAG-3′ and Reverse: 5′-ATTTGDCCACAGTGDATACCA-3′) were used Li et al. (2013). Reactions were performed in a 20 μl volume containing 1 μl of DNA, 0.4 μM of each primer and 10 μl of TB Green Premix Ex Taq II (TliRNaseH Plus, Takara, Tokyo, Japan). Thermal cycling conditions for qPCR reactions were: 95°C for 1.2 min; 45 cycles of 15 s at 95°C; 1 min at 56°C; 15 s at 95°C. Melting curves were run between 60 and 95°C. The specificity of the amplification product was verified by melting curve analysis. The R^2^ for standard curves of Bacillariophytes and Pelagophyceae were 0.996 and 0.992, respectively, whereas efficiencies were 105.0 and 167.2%, respectively. Plasmid DNA with inserted *rbc*L gene was measured with a NanoDrop 2000 spectrophotometer (Thermo Fisher Scientific, Wilmington, DE, United States) and used as standards. Copy numbers of the *rbc*L gene were then calculated based on the concentration of plasmid DNA and amplicon size as previously described (Okano et al., 2004; He et al., 2007).

### Data Processing and Statistical Analysis

The raw sequence data was transformed into sequence reads by base calling. These reads were stored in FASTQ files which contained raw sequence data with respective sequencing quality. The raw sequence data were quality-filtered and analyzed through open source software QIIME (v1.8) (Caporaso et al., 2010). Further, paired reads were merged into full-length sequences by FLASH software (v1.2.7) and filtered with Trimmomatic software (v0.33). Sequences meeting the following three criteria were included in downstream analyses: (1) sequences with precise primers and bar-codes; (2) quality score > 30; and (3) sequences > 200 bp in length. The software package USEARCH (v1.8) was then used to further eliminate erroneous and chimeric sequences. After removing non-*rbc*L sequence reads, sequences were clustered into operational taxonomic units (OTUs) at a 97% similarity level using UCLUST (v1.2.22). Low-abundance OTUs (fewer than 2 reads, including singletons) were excluded from the subsequent analyses (Dickie, 2010). The remaining high-quality sequences were queried against the GenBank database at NCBI using local BLASTn. The MEGAN program (Huson et al., 2007) was used to assign BLAST hits to taxa in the NCBI database. The sequences obtained from this study have been deposited in NCBI Sequence Read Archive with Accession No. SUB4422106.

Richness estimator (Chao1) and diversity indices (Shannon and Simpson) were calculated using QIIME v1.8. Gene abundances were calculated and plotted with Golden Software Surfer 11 (Golden Software, United States). Redundancy analysis (RDA) (Canoco, v 4.5) was performed to examine correlations between OTU abundance and environmental parameters. The number of sequences in each OTU was log transformed before conducting RDA. Pearson Correlation analysis (PAST, Version-3.23, Natural History Museum, University of Oslo, Norway) was performed for testing significant differences between gene copy numbers of diatoms and pelagophytes with environmental parameters including nutrients, chlorophyll, temperature, and salinity. Pearson correlation analysis was also used for to obtain significant difference between morphologically identified phytoplankton and environmental variables. The differences of various environmental parameters as well as qPCR detected *rbc*L gene abundances of chromophytic phytoplankton between Northern BOB and Southern BOB were evaluated by *t*-test using Excel (Command TTEST, two-tailed).

## Results

### Environmental Parameters

**FIGURE 2:**
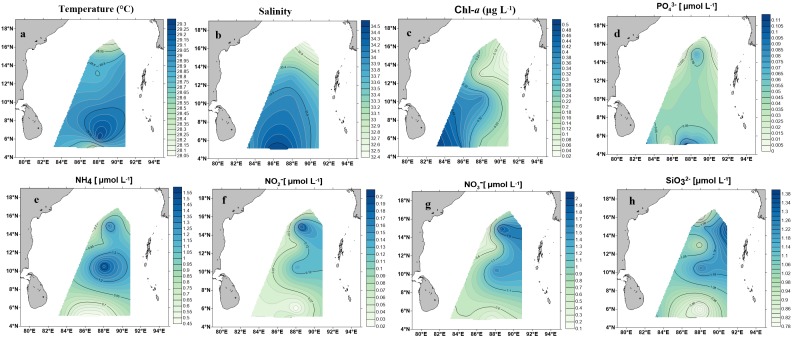
Contour maps demonstrating environmental parameter measurements on the surface water in the BOB. **(a)** Temperature (°C), **(b)** salinity, **(c)** Chl-*a* (μg/L), **(d)** phosphate (${\text{PO}}_{4}^{3-}$), **(e)** ammonia (${\text{NH}}_{4}^{+}$), **(f)** nitrate (${\text{NO}}_{2}^{-}$), **(g)** nitrite (${\text{NO}}_{3}^{-}$), **(h)** silicate (${\text{SiO}}_{3}^{2-}$).

Environmental parameters are listed in Table 1 and detailed contour maps for environmental parameter gradients are presented in Figure 2. Phosphate, ammonium, nitrate, nitrite, and silicate concentrations ranged from 0.09 to 0.574 μM (average 0.048 ± 0.03 μM), 0.457 to 1.571 μM (average 0.86 ± 0.30 μM), 0.021 to 0.200 μM (average ± 0.78 μM), 0.021 to 0.2 μM (average 0.076 ± 0.05 μM) and 0.790 to 1.379 μM (average 1.03 ± 0.18 μM), respectively (Table 1). Among all measured nutrients, phosphate (*p* < 0.01) was fairly in low concentrations at all stations and the highest concentration was observed at station BOB 10 (Figure 2). In contrast, ammonium, nitrate, and silicate concentrations were generally higher in BOB than stations near the Equator region (*p* > 0.05) (Figure 2b–d). Surface water temperature in BOB fairly varied from northern to southern near the Equator with an average of 28.6 ± 0.34°C (*p* > 0.05) (Table 1). Higher salinities were observed in the southern region, which ranged from 32.38 (BOB-1) to 34.68 (BOB-8) with an average of 33.5 ± 0.69 (*p* < 0.01). Geographically, the BOB contained lower surface salinity and temperature than the equator region because of freshwater input from adjacent rivers in the northern BOB and massive rainfall during the prevailing monsoon. Chl-*a* concentrations ranged from 0.017 to 0.560 μg L^−1^ with an average of 0.24 ± 0.16 μg L^−1^ (Table 1). Chl-*a* concentrations showed a similar trend as salinity and temperature: increased from northern to the southern region of the BOB (*p* < 0.05) (Figure 2a–c).

**Table 1:** Temperature (Temp), salinity (S), chlorophyll *a* (Chl-*a*) concentration and dissolved inorganic nutrients (${\text{PO}}_{4}^{3-}$, ${\text{NH}}_{4}^{+}$, ${\text{NO}}_{3}^{-}$, ${\text{NO}}_{2}^{-}$, and ${\text{SiO}}_{3}^{2-}$) measured on the surface layer at the survey stations.

Stations	Longitude (°E)	Latitude (°N)	Temperature (°C)	Salinity	Chl-*a* (μg L^−1^)	${\text{PO}}_{4}^{3-}$ (μM L^−1^)	${\text{NH}}_{4}^{+}$ (μM L^−1^)	${\text{NO}}_{3}^{-}$ (μM L^−1^)	${\text{NO}}_{2}^{-}$ (μM L^−1^)	${\text{SiO}}_{3}^{2-}$ (μM L^−1^)
BOB-1	89.499	17.014	28.03	32.38	0.167	0.023	0.821	0.671	0.029	1.000
BOB-2	90.997	14.998	28.92	32.66	0.017	0.000	0.779	1.595	0.121	1.379
BOB-3	88.007	15.489	28.35	33.00	0.195	0.019	0.721	0.467	0.050	0.768
BOB-4	88.497	15.001	28.72	33.08	0.176	0.074	1.364	1.980	0.200	1.190
BOB-5	87.001	15.005	28.77	33.04	0.277	0.000	0.564	0.111	0.042	1.129
BOB-6	87.964	12.987	28.79	33.77	0.093	0.045	0.943	0.459	0.064	0.939
BOB-7	87.998	10.509	28.93	34.03	0.391	0.048	1.529	1.562	0.136	1.258
BOB-8	91.003	05.004	28.82	33.68	0.150	0.045	0.843	0.628	0.086	1.020
BOB-9	87.994	05.997	29.30	34.26	0.222	0.058	0.629	0.502	0.021	0.790
BOB-10	87.498	05.000	28.34	34.42	0.228	0.110	0.471	0.306	0.071	0.891
BOB-11	85.610	05.000	28.44	34.38	0.564	0.081	0.850	0.578	0.050	0.888
BOB-12	83.000	05.003	28.88	33.78	0.500	0.081	0.850	0.578	0.050	1.107

### Community Composition and Phylogenetic Analysis of Chromophytic Phytoplankton

A Total of 385,077 raw sequences were generated from our samples and 152,556 clean sequences were included in our downstream analysis (Table 2). The rarefaction curves were shown in Supplementary Figure 1, and most curves were plateaued. A total of 662 OTUs were retrieved after clustering at a 97% similarity level (Table 2). Shannon-Weiner H index was calculated for all the stations and the highest value occurred at station BOB-12 (6.24) whereas the lowest value was recorded at BOB-1 (3.24) (Table 2). OTU richness was calculated using non-parametric Chao1 estimator. The highest Chao1 value was observed at BOB-2 whereas the lowest Chao1 value was recorded at BOB-10 (Table 2). The species richness and Good’s coverage were also listed in Table 2.

**Table 2:** Summary of OTU numbers and diversity indices for *rbc*L sequences recovered from the BOB.

	Stations											
	BOB-1	BOB-2	BOB-3	BOB-4	BOB-5	BOB-6	BOB-7	BOB-8	BOB-9	BOB-10	BOB-11	BOB-12
No. of OTUs	298	350	293	346	262	286	323	323	324	280	313	332
Raw tags	37992	24636	20597	20673	33122	32092	34223	41633	26069	45731	42549	26760
Final Tags	12713	12713	12713	12713	12713	12713	12713	12713	12713	12713	12713	12713
Species richness	290	343	288	339	254	278	315	316	316	275	304	328
Chao1	335.08	401.51	324.67	385.54	348.98	361.6	400.03	378.56	374.69	309.02	369.23	359.4
Good’s Coverage	0.99	0.99	1	0.99	0.99	0.99	0.99	0.99	0.99	1	0.99	1
Shannon-Weiner	3.25	4.94	4.32	4.53	4	3.83	4.13	4.6	3.99	4.64	4.14	6.24

**FIGURE 3:**
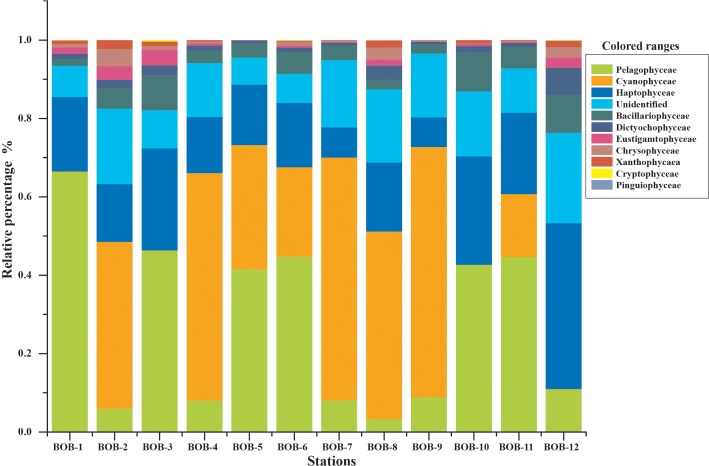
Community structure of chromophytic phytoplankton at class level based on *rbc*L gene sequences.

**FIGURE 4:**
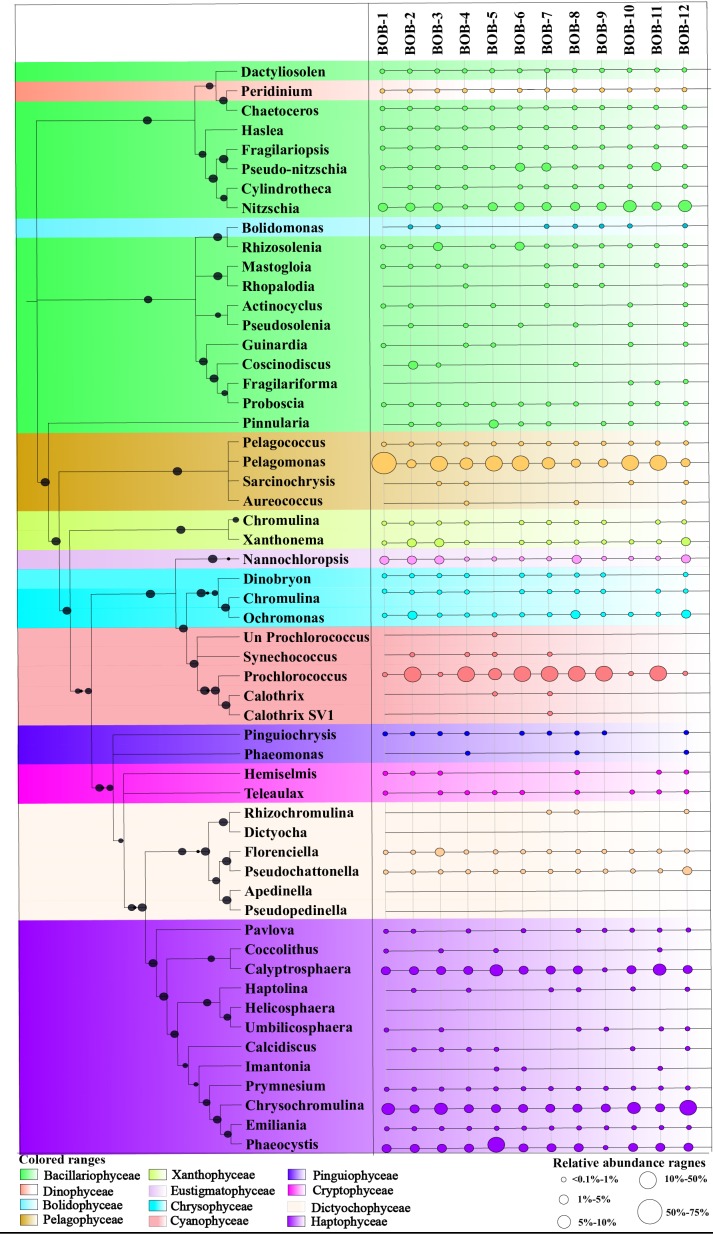
A neighbor-joining phylogenetic tree constructed based on *rbc*L amino acid sequences. The topology of the tree was inferred from 1000 bootstrap resampling, and bootstrap values greater than 50% were labeled with black dots at branches.

All major chromophytic phytoplankton groups containing form ID *rbc*L genes such as Bacillariophyceae, Haptophyceae, Pelagophyceae, Chrysophyceae, Cryptophyceae, Dictyochophyceae, Cyanophyceae, Eustigamatophyceae, Xanthophyceae, and Pinguiophyceae as well as some rarely occurring groups were detected. The most abundant classes and genera were shown in Figure 3, 4, respectively. Bacillariophyceae-like *rbc*L sequences were recovered from most of the stations in the study area while Pelagophyceae, Haptophyceae, and Cyanophyceae were observed to be dominant (Figure 4).

A neighbor-joining phylogenetic tree based on *rbc*L gene amino acid sequences was constructed and shown in Figure 4. Bacillariophyceae contained the most diverse sequences and 19 OTUs were found: *Nitzschia* was the dominant genus recovered at stations BOB-10 and BOB-12 followed by *Pseudo-nitzschia* and *Rhizosolenia* (Figure 4). Notably, *Bolidomonas*, a genus containing picoplanktonic flagellated algae, was also recovered from station BOB-12. *Peridinium* was another genus that occurred at station BOB-12, which is symbiotic with diatoms and was discussed further in this study. Four genera were detected within class Pelagophyceae (4 OTU), and among them, *Pelagomonas* was dominant at most of the stations (e.g., BOB-1) whereas lowest abundance was recovered from station BOB-8. Other genera clustered in Pelagophyceae were *Pelagococcus*, *Aureococcus*, and *Sarcinochrysis*. Regarding Haptophyceae, it was represented by 12 OTUs which were dominated by genera *Chrysochromulina* and *Calyptrosphaera*. *Chrysochromulina* was dominant at station BOB-12 whereas *Calyptrosphaera* was dominant at BOB-5. Among class Cyanophyceae (5 OTUs), *Prochlorococcus* was the only dominant genus in recovered sequences. Further, Dictyochophyceae clade contained 6 OTUs and was represented by *Apedinella* at station BOB-12. Other classes included Cryptophyceae (2 OTUs), Eustigmatophyceae (1 OTU), Xanthophyceae (2 OTUs), and Chrysophyceae (3 OTUs) (Figure 3).

### Quantification of *rbc*L Genes From Diatoms (Bacillariophytes) and Pelagophytes

**FIGURE 5:**
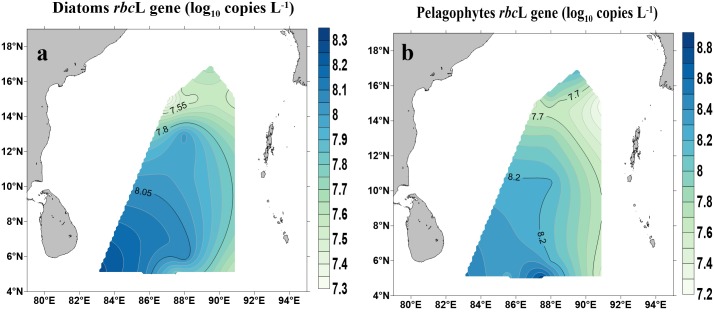
Abundance of *rbc*L genes for diatoms (Bacillariophyceae) **(a)** and Pelagophytes (Pelagophyceae) **(b)** in the BOB.

Abundances of *rbc*L genes (copies L^−1^) for diatoms (Bacillariophytes) and pelagophytes and their spatial distribution are shown in Figure 5. Both diatoms and pelagophytes showed similar spatial patterns with higher copy numbers occurred near the Equator and lower in the northern BOB (*p* < 0.01) (Figure 5). The copy numbers of *rbc*L genes for diatoms ranged from 40.4 ± 5.46 × 10^4^ copies L^−1^ (BOB-5) to 30.8 ± 4.61 × 10^5^ copies L^−1^ (BOB-12). Pelagophyceae *rbc*L gene copy numbers varied across stations: the highest number (11.4 ± 3.33 × 10^6^) copies L^−1^ was found near the Equator (BOB-10) whereas the lowest was recorded (32.2 ± 4.79 × 10^4^ copies L^−1^) at station BOB-2.

### Morphological Identification of Phytoplankton and Quantification

**FIGURE 6:**
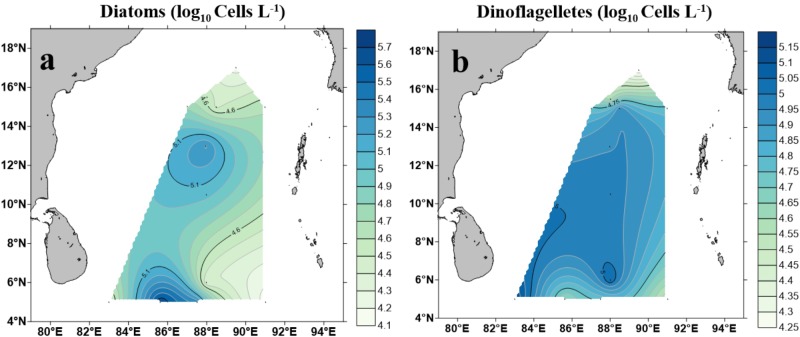
Cell counts of diatoms (Bacillariophyceae) **(a)** and dinoflagellates **(b)** in the BOB based on microscopic identification.

A total of 96 taxa of phytoplankton (morphologically identified phytoplankton) were identified through microscopic observation. Dinoflagellates and diatoms were observed to be the most diverse and abundant groups while cyanobacteria were identified as less diverse but predominant in some stations of the study area (e.g., BOB-9 and BOB-11) (Supplementary Table 1). Contour maps for abundance and spatial distribution of diatoms and dinoflagellates (identified using microscope) at BOB are shown in Figure 6. Diatoms ranged from 1.4 × 10^4^ cells L^−1^ (BOB-8) to 4.84 × 10^5^ cells L^−1^ (BOB-11), Whereas dinoflagellates, ranged from 1.8 × 10^4^ cells L^−1^ (BOB-1) to 1.42 × 10^5^ cells L^−1^ (BOB-12) (Supplementary Table 1). Compared to other groups, Chrysophyta was less diverse and abundant, and its abundance ranged from 8 × 10^3^ cells L^−1^ (BOB-7, BOB-8, and BOB-12) to 1.6 × 10^4^ cells L^−1^ (BOB-4). Overall through microscopic identification, we identified major groups such as diatoms, dinoflagellates, cyanophytes, and chrysophytes. Among these, dinoflagellates were barely detected from our molecular characterization (Figure 4). In addition, *Trichodesmium thiebaultii* was successfully identified through microscopy as a major cyanobacterial group which was missing from molecular characterization. But due to the small size of picocyanobacterial cells (e.g., *Prochlorococcus* and *Synechococcus*) very likely we missed them in our microscopic identifications.

### Correlations Between Phytoplankton Quantified Through Morphological Observation and qPCR Detected Gene Copy Number With Environmental Variables

**FIGURE 7:**
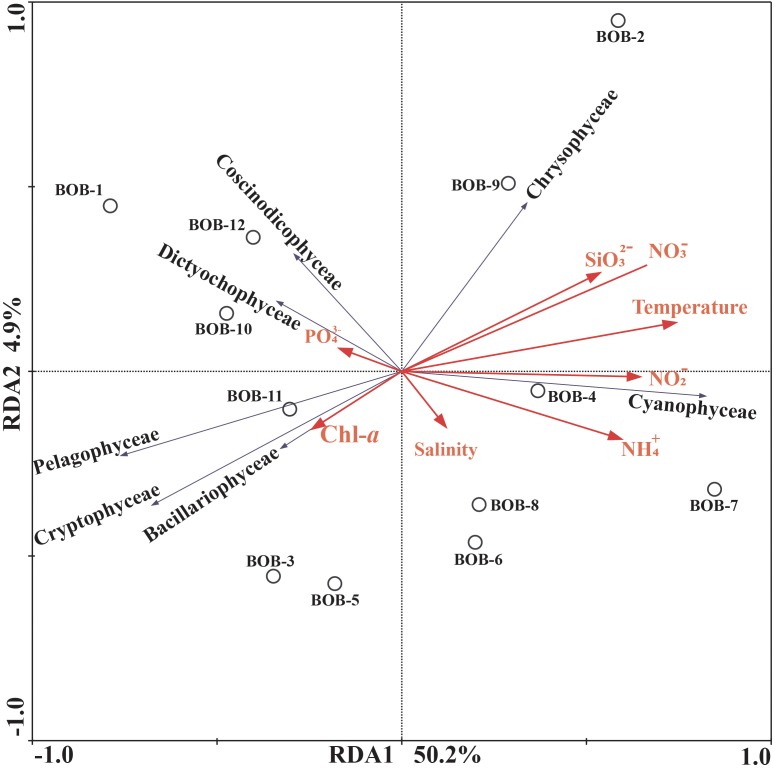
Redundancy analysis (RDA) for *rbc*L gene-based chromophytic phytoplankton community distribution and environmental factors. The black arrows represented different classes of phytoplankton whereas environmental variables were shown with red arrows. Correlation between environmental variables (Temperature, Salinity, ${\text{PO}}_{4}^{3-}$, Chl-*a*, ${\text{NH}}_{4}^{+}$, ${\text{NO}}_{3}^{-}$, ${\text{NO}}_{2}^{-}$, and ${\text{SiO}}_{3}^{2-}$) and RDA axes were shown by both length and angle of arrows. The two RDA axes explained 55.1% of total variation.

Correlations between chromophytic phytoplankton communities and the associated environmental factors are shown by RDA plot (Figure 7). The environmental factors in the first two axes explained 55.1% of the total variances. Our RDA analysis indicated that among all environmental variables, temperature (*p* < 0.05) was the major factor determining the distribution of chromophytic phytoplankton (Figure 7). In addition, RDA also showed correlations of other environmental variables with chromophytic phytoplankton groups. For instance, Chrysophyceae positively responded to ${\text{NO}}_{3}^{-}$, temperature, and ${\text{SiO}}_{3}^{2-}$. Further, Dictyochophyceae and Coscinodiscophyceae responded to ${\text{PO}}_{4}^{3-}$ while Cyanophyceae showed significant positive relationships with temperature, ${\text{NO}}_{2}^{-}$ and ${\text{NH}}_{4}^{+}$ (Figure 7). In contrast, Cryptophyceae, Pelagophyceae, and Bacillariophyceae were negatively correlated to ${\text{NO}}_{3}^{-}$, ${\text{SiO}}_{3}^{2-}$ and temperature. Moreover, Pearson’s correlations of *rbc*L gene abundances and various physicochemical parameters were listed in Table 3. Diatoms and pelagophytes positively correlated to Salinity (*p* < 0.01), Chl-*a* (*p* < 0.01), ${\text{PO}}_{4}^{3-}$ (*p* < 0.01), and ${\text{NO}}_{2}^{-}$ (*p* < 0.05). Diatoms also showed a significantly positive relationship with temperature but Pelagophytes were negatively correlated with temperature. Negative correlations were also observed between ${\text{SiO}}_{3}^{2-}$ with diatoms and pelagophytes. Further, Pearson’s correlation matrix of physicochemical parameters and morphologically identified phytoplankton indicated diatoms exhibited positive correlations with salinity and Chl-*a*, but a negative relationship with ${\text{NH}}_{4}^{+}$ (Table 4). As a group present in morphological identification but not recovered in our molecular characterization, dinoflagellates showed positive correlations with all of the physicochemical parameters besides ${\text{NO}}_{3}^{-}$_._ Cyanophyta was positively correlated with temperature and salinity but negatively correlated with ${\text{PO}}_{4}^{3-}$, ${\text{NH}}_{4}^{+}$, ${\text{NO}}_{3}^{-}$, and ${\text{SiO}}_{3}^{2-}$.

**Table 3:** Pearson correlation matrix of qPCR-detected gene copy numbers and physicochemical parameters.

	Temperature	Salinity	chl-*a*	${\text{PO}}_{4}^{3-}$	${\text{NH}}_{4}^{+}$	${\text{SiO}}_{3}^{2-}$	${\text{NO}}_{3}^{-}$	${\text{NO}}_{2}^{-}$
Diatoms	0.367^∗∗^	0.635^∗∗^	0.646^∗∗^	0.544^∗∗^	0.066	−0.212^∗^	−0.183	−0.285^∗^
Pelagophytes	−0.288^∗^	0.544^∗∗^	0.392^∗∗^	0.742^∗∗^	−0.304^∗∗^	−0.305^∗∗^	−0.334^∗∗^	−0.249^∗^

*^∗^p < 0.05; ^∗∗^p < 0.01.*

**Table 4:** Pearson correlation matrix of physicochemical parameters and morphologically identified phytoplankton in the BOB (Temp: Temperature, Sal: Salinity, Dino: Dinoflagellates, Chr: Chrysophyta, Cyan: Cyanophyta).

	Temp	Sal	Chl-*a*	${\text{PO}}_{4}^{3-}$	${\text{NH}}_{4}^{+}$	${\text{NO}}_{3}^{-}$	${\text{NO}}_{2}^{-}$	${\text{SiO}}_{3}^{2-}$	Dino	Diatoms	Chr
**Temp**											
Sal	0.41^∗∗^										
Chl-*a*	0.14	0.51^∗∗^									
${\text{PO}}_{4}^{3-}$	0.01	0.56^∗∗^	0.26^∗^								
${\text{NH}}_{4}^{+}$	0.16	−0.16	−0.00	0.14							
${\text{NO}}_{3}^{-}$	0.23^∗^	−0.32^∗∗^	−0.26^∗^	−0.17	0.55^∗∗^						
${\text{NO}}_{2}^{-}$	0.14	−0.14	−0.19	0.18	0.74^∗∗^	0.84					
${\text{SiO}}_{3}^{2-}$	0.22^∗^	−0.39^∗∗^	−0.08	−0.19	0.54^∗∗^	0.77^∗∗^	0.65^∗∗^				
Dino	0.71^∗∗^	0.45^∗∗^	0.33^∗∗^	0.45^∗∗^	0.30^∗∗^	0.06	0.25^∗^	0.20^∗^			
Diatoms	−0.02	0.56^∗∗^	0.26^∗∗^	0.08	−0.21^∗∗^	−0.19	−0.02	−0.14	0.16		
Chr	0.29^∗^	0.24^∗∗^	0.46^∗∗^	0.25^∗^	0.44^∗∗^	0.09	0.16	0.31^∗∗^	0.23^∗^	−0.27^∗^	
Cya	0.26^∗^	0.27^∗∗^	0.09	−0.35^∗∗^	−0.48^∗∗^	−0.49^∗∗^	−0.54^∗∗^	−0.61^∗∗^	0.08	0.33^∗∗^	−0.48^∗∗^

*^∗^p < 0.05; ^∗∗^p < 0.01.*

## Discussion

### Phylogenetic Characterization and Global Distribution of Chromophytic Phytoplankton in the BOB

It is noticeable that few effort and studies have been conducted to characterize chromophytic phytoplankton communities in the BOB, especially at the molecular level. Recent studies primarily focused on phylogenetic analyses based on 16S rRNA genes in both coastal and open oceans as well as in oxygen minimum zone (OMZ) of the BOB (Bristow et al., 2017; Choi et al., 2017; Menezes et al., 2018; Rajpathak et al., 2018). To our best knowledge, no studies have investigated community structures of chromophytic phytoplankton based on functional genes such as *rbc*L. This study serves as the first report on the molecular population structure of photosynthetic organisms in BOB and provides baseline data for future studies.

Our high throughput sequencing data revealed major chromophytic groups including Bacillariophyceae, Haptophyceae, Pelagophyceae, Cyanophyceae and less abundant groups such as Cryptophyceae, Chrysophyceae, Eustigmatophyceae, Xanthophyceae, Dictyochophyceae, and Pinguiophyceae. These results were consistent with the results from previous investigations carried out in different environments, suggesting great homogeneity of chromophytic phytoplankton occurring in most marine ecosystems of the world (Pichard et al., 1993; Paul et al., 1999; Bhadury and Ward, 2009; Samanta and Bhadury, 2014; Li et al., 2016). The most diverse and predominant chromophytic phytoplankton group retrieved in our study, diatoms (Bacillariophyceae) are globally distributed and they constitute 20% of the global primary production (Nelson et al., 1995; Round et al., 2007). Undoubtedly, diatoms are the most successful algae present from freshwater to seawater, and from the surface to benthic habitats (Werner, 1977; Mann and Droop, 1996). Since there is no characterization of diatoms at the molecular level in the BOB, a few studies that performed morphological characterizations supported our observation of diversity and abundance of Bacillariophyceae (Paul et al., 2007; Boonyapiwat et al., 2008; Li et al., 2012; Manjumol et al., 2018).

Our phylogenetic analysis also revealed that *Bolidomonas* (*Triparma*)-like *rbc*L sequences shared high similarity (94%) with Bacillariophyceae *rbc*L sequences (Figure 3). With isolated *Bolidomonas* strains and by comparing nuclear, plastidial and mitochondrial gene markers, Ichinomiya et al. (2016) have combined *Bolidomonas* into *Triparma* and included both of them into *Parmales* (Bolidophyceae). The phylogenetic assessment suggested that *Parmales* (Bolidophyceae) was closely related to diatoms (Bacillariophyceae). The study also demonstrated that Bolidophyceae were ubiquitously distributed but only constituted a minor component of the phytoplankton community (Ichinomiya et al., 2016). Nonetheless, this is the first record of *Bolidomonas* (*Triparma*) from the BOB. Similarly, we recovered *Peridinium* (Dinophyceae)-like *rbc*L sequences which were also clustered with Bacillariophyceae in the phylogenetic tree (Figure 3). By a systematic investigation of SSU rDNA sequences, Inagaki et al. (2000) succeeded in recovering the closest sister relationship between two dinoflagellates *Peridinium balticum* and *Peridinium foliaceum*, and it was suggested that the ancestors of these two dinoflagellates engulfed a pennate diatom, and therefore showed a close affinity to diatom *rbc*L gene sequences.

Pelagophyceae was another dominant group observed in our chromophytic phytoplankton analysis. Among this class, *Pelagomonas calceolata* has been referred to as an open ocean species (Saunders et al., 1997). From the discovery of *Pelagomonas calceolate* to recent studies based on 18S rRNA genes, this species has been documented a global distribution in surface or subsurface waters in North Pacific Ocean (Andersen et al., 1993), South Pacific Ocean (Moon-van der Staay et al., 2000), and the Mediterranean Sea (Dìez et al., 2001). *Pelagomonas* sequences were also recovered from the surface layer in the central North Pacific gyre of the Global Ocean Survey (Rusch et al., 2007). More recently, a study in the subarctic Pacific Ocean obtained *Pelagomonas* sequences from deep sediments, suggesting a wide vertical distribution of *Pelagomonas* from the euphotic zone to deep oceans (Worden et al., 2012). In this study, our functional gene (*rbcL*) analysis verified the presence and dominance of *Pelagomonas* species at BOB. This is the first record of *Pelagomonas calceolate* in BOB.

Further, considerable Haptophyceae sequences were retrieved from the BOB. Previous observations have demonstrated that these picoplanktonic flagellates are successful cosmopolitan picophytoplankton that inhabits a great variety of environments ranging from freshwater to marine habitats (Thomsen, 1994; Jordan and Chamberlain, 1997; Jordan et al., 2004; Edvardsen et al., 2011). Within Haptophyceae, most sequences were closely related to *Chrysochromulina*, a ubiquitous genus that has been recovered in European waters (Parke et al., 1955; Wujek, 1986), South Africa, Australian coasts (Hallegraeff, 1983; Pienaar and Bandu, 1984), and in the Arctic region of Canada and Alaska as well (Manton, 1978; Kling and Kristiansen, 1983).

### Influence of Physicochemical Parameters on the Spatial Distribution of Chromophytic Phytoplankton

The BOB is a semi-enclosed tropical sea, which is highly influenced by seasonal monsoons. In addition to the freshwater influx from riverine discharge (Ganges River), monsoons bring a significant amount of fresh water through high precipitation. Moreover, ocean currents [e.g., East India Coastal Current (EICC) and Southwest Monsoon Current (SMC)] and eddies are able to erode stratification as well as bring upwelling nutrients to surface waters (Gomes et al., 2000; Schott and Mccreary, 2001; Shankar et al., 2002). Such physical forces change the surface physicochemical parameters and consequently affect the composition and productivity of chromophytic phytoplankton. Our RDA analysis, qPCR results and microscopic cell counts confirmed that the spatial distribution of phytoplankton responded to changes in physicochemical parameters such as temperature and nutrients (Figure 7 and Tables 3, 4). Abundance and community structure of diatoms (Bacillariophyceae) responded to temperature but no consistent results were observed among different analyses. RDA analysis indicated that diatoms were negatively related to temperature (Figure 7), qPCR results indicated a positive relationship (Table 3), while no significant relationship was found between microscopic cell counts vs. temperature (Table 4). In general, the temperature is a key environmental parameter driving phytoplankton distribution and activity (Patrick, 1948; Montagnes and Franklin, 2001). Inconsistent results from this study suggested that in addition to temperature, other environmental parameters also impact on diatom abundance and distribution at BOB. Smaller phytoplankton groups with large surface area to volume ratio can outcompete diatoms during nutrient limiting conditions and warm water temperatures (Read et al., 2014). Moreover, a laboratory-grown culture experiment has shown that diatoms were not an exception to the rule that cell size reduces with increase in temperature (Montagnes and Franklin, 2001), where size-selective grazing may become a limiting factor for phytoplankton (Peter and Sommer, 2013). Pelagophyceae showed similar distribution patterns and most of the genera recovered in this study have been reported being dominant in warm surface water (Agawin et al., 2000; Mitbavkar et al., 2015).

Another major chromophytic phytoplankton group responding to warm temperature was Cyanobacteria (mainly *Prochlorococcus*). *Prochlorococcus* is ubiquitously distributed from 40°N to 40°S, and from the surface to 150 m deep (Flombaum et al., 2013). Recent research based on functional genes (*rbc*L) verified the global distribution (involving Atlantic, Pacific, and the Indian Ocean) of *Prochlorococcus* and confirmed their distribution from the surface to DCM (Fernández-Pinos et al., 2017). *Prochlorococcus* generally proliferate in tropical and subtropical oligotrophic waters which are influenced by seasonality (Campbell et al., 1994; Campbell et al., 1997; Partensky et al., 1999). Our RDA analysis showed a strong positive correlation of Cyanophyceae with temperature. Different strains of *Prochlorococcus* responded differently to light availability and temperature and their population size declined below 15°C (Johnson et al., 2006). Our study was conducted during prevailing monsoon, which brought dense clouds and limited the availability of sunlight, and therefore influenced growth of Cyanobacteria especially *Prochlorococcus*. Further, we could not neglect the emerging OMZ in BOB and its impact on the distribution of Cyanobacteria. Recent studies have concluded that BOB, currently referred to as weak OMZ, is in the verge of becoming the next OMZ (Bristow et al., 2017). At South Pacific OMZ, *Synechococcus* were abundantly present in the surface oxic waters while *Prochlorococcus* were present in the subsurface low-oxygenated water (Lavin et al., 2010). Contrastingly, we found more prevalence of *Prochlorococcus* and very low abundances of *Synechococcus* sequences at surface water. This probably can be linked to a developing OMZ in the BOB. Large riverine network successfully transported organic matter to the northern part of the BOB that enhanced microbial respiration and resulted in less oxygen and nitrogen-based oxidants thereby producing weak OMZ and suppressed denitrification (Al Azhar et al., 2017; Bristow et al., 2017). Investigating interactions between *Prochlorococcus* and *Synechococcus* and their distribution at depths (including OMZs) will provide insightful information on primary producers in the BOB and is greatly needed. Although Cyanobacteria mostly favor warm and oligotrophic waters, they are also present in coastal areas where the nutrient is not a limiting factor. For example, they were found in coastal areas in Plume of the Rhone river, Mediterranean Sea (Veldhuis and Kraay, 1990) and Suruga Bay, Japan (Shimada et al., 1995). Lastly, the co-occurrence of Cyanobacteria with other chromophytic phytoplankton sequences suggests the existence of a possible symbiotic relationship. Jyothibabu et al. (2006) explained that during a spring inter-monsoon, nitrate limitation was pronounced at the surface water of the BOB and Cyanobacteria would benefit from symbiotic relationships with other algae or dinoflagellates. In fact, symbiotic relationships between Cyanobacteria and diatoms have been previously explored within studies undertaken in different parts of Indian Ocean such as the BOB, the Arabian Sea and the Andaman Sea (Kulkarni et al., 2010; Jabir et al., 2013; Manjumol et al., 2018).

In the BOB, possible nutrients sources originated from two regions: (1) northern riverine runoffs in northern BOB and (2) Southwest upwelling region near the Equator. Most abundant chromophytic phytoplankton groups including Pelagophyceae, Haptophyceae, and Bacillariophyceae showed higher abundances at both northern BOB and near the Equator region (Figure 3, 4), suggesting that high nutrient concentration likely support growth of these chromophytic phytoplanktons. A previous study based on microscopic identification of phytoplankton found a high abundance of phytoplankton in northern BOB and deduced nutrient discharging into the BOB from riverine runoff as the factor driving phytoplankton distribution (Boonyapiwat et al., 2008). Meanwhile, during the northeast monsoon, the southwestern part of the BOB experiences cyclonic gyre. The cyclonic gyre is primarily forced by Ekman pumping and positive wind stress that result in upwelling. High Chl-*a* concentrations were observed in southern BOB (between 11 and 13°N) (Gomes et al., 2000; Jyothibabu et al., 2015) and Vinayachandran and Mathew (2003) has concluded that it is likely due to high nutrients from gyre upwelling water. Moreover, we observed higher diversity of chromophytic phytoplankton in the southern stations near the Equator region (e.g., BOB 12). Thus, we hypothesized that increased availability of nutrients from riverine runoffs along Northern BOB and upwelling water at Southwest BOB play important roles in manipulating structure and distribution of chromophytic phytoplankton.

### Phytoplankton Identified by High Throughput Sequencing vs. Microscopic Morphology

Morphological identification of phytoplankton is very challenging and extensive expertise is needed. Through microscopy analysis, we observed diatoms, dinoflagellates, chrysophytes, and cyanophytes were the major groups occurred in the BOB surface waters. Most of the previous studies in characterizing phytoplankton through morphological identification were restricted to coastal zones of the BOB, and very limited investigations were carried out in open oceans at the BOB (Paul et al., 2007). Boonyapiwat et al. (2008) conducted a survey during northwest monsoon and the results showed a high abundance of diatoms occurred in the northern BOB. Most sampling stations in this study were located in the coastal zone and northern BOB, and the abundance of diatoms was probably influenced by riverine nutrient input (Boonyapiwat et al., 2008). In our study, we found a higher abundance of phytoplankton in the southern open ocean zone of the BOB which was likely impacted by SMC current and upwelling near Sri Lanka (Figure 5–7).

When we compared chromophytic phytoplanktons sequences using high throughput sequencing to qPCR assays on morphologically identified phytoplankton, a significant difference was observed between gene copy numbers detected by qPCR vs. direct microscopic counts (Figure 5, 6) in diatoms. Specifically, morphologically identified diatoms ranged between 1.8 × 10^4^ and 4.8 × 10^5^ cells L^−1^ whereas qPCR derived diatom (Bacillariophyceae) gene copy numbers showed as low as 4.04 × 10^5^ to as high as 3.08 × 10^6^ copies L^−1^. One possible explanation was each picoplanktonic diatom cell contained multiple copies of *rbc*L genes (Pichard et al., 1993). For instance, in addition, two copies of *rbc*L genes spanning on their genomes, diatom cells may contain up to 2000 chloroplast-encoding *rbc*L genes (Douglas, 1988). Therefore, the discrepancy observed between gene copies and morphologically counted cell numbers could be justified as due to the presence of multiple copies of *rbc*L genes in diatom cells.

Further, we observed that some species and groups of chromophytic phytoplankton were missing from our molecular characterization. For instance, *Trichodesmium thiebaultii* was identified as the dominant cyanobacterial species under microscopic counts but it was completely missing in our high throughput sequencing analysis (Figure 3 and Supplementary Table 1). This could most likely be attributed to the bias introduced by the primers used in our study (form ID *rbcL*) for PCR amplification. Evolution of RuBisCO-encoding *rbc*L genes gave rise to different forms used for identifying different groups of phytoplankton. Form IA, IB, IC, and ID encode green and red like RuBisCOs and they exist in different groups of phytoplankton (Watson and Tabita, 1997). Form IB occurred in green plants lineage and some Cyanobacteria, whereas form ID was widely distributed in chromophytic algal groups such as diatoms, prymnesiophytes, pelagophytes, and chrysophytes (Paul et al., 1999). RuBisCO in *Trichodesmium* was likely encoded by *rbc*L form IB (Paul et al., 1999; Chen et al., 2004; Tomitani et al., 2006) and was, therefore, missing from our PCR amplification. Our morphological observation also identified dinoflagellates as another major phytoplankton group (Supplementary Table 1) which agreed with a previous study (Madhupratap et al., 2003). Previous studies carried out in the Chesapeake Bay (MD, United States), the Jiaozhou Bay (Qingdao, China), and the Bay of Biscay (western coast of France) have shown that morphologically identified diatoms, as well as dinoflagellates, were consistently dominant and diverse in different ecosystems (Marshall and Lacouture, 1986; Shen, 2001; Smythe-Wright et al., 2014). However, dinoflagellates were under-represented in our molecular analysis. This could perhaps be explained by Morse’s finding (1995) that RuBisCO in dinoflagellates was possibly encoded by RuBisCO form II instead of form I. Also, dinoflagellates RuBisCO were perhaps encoded by genomic DNA and not by chloroplast DNA (Morse et al., 1995). A recent study undertaken to investigate ID *rbc*L gene diversity spanning seven different seas around the world did not recover dinoflagellates sequences (Samanta and Bhadury, 2016). As a single gene marker, form ID *rbc*L gene covers bacteria (Alpha, Beta, and Gamma-proteobacteria) and micro-phytoplankton such as Bacillariophyceae, Haptophyceae, Pelagophyceae, Cryptophyceae, and Bolidiophyceae (Samanta and Bhadury, 2016), but may have missed other groups including dinoflagellates and cyanobacteria. Our results highlight the necessity of a combination of molecular analysis targeting *rbc*L genes and microscopic detection in examining phytoplankton composition and diversity.

## Conclusion

Our observations on chromophytic phytoplankton demonstrate the diversity of these primary producers and their spatial distribution in the BOB. Environmental variables such as temperature and nutrients were the most significant factor which influenced the community structure of chromophytic phytoplankton. Primarily we observed: (1) greater diversity and community composition of chromophytic phytoplankton in the BOB that has never been studied before, (2) morphological identification in combination with molecular characterization provided insights of overall community compositions, and (3) spatial distribution of chromophytic phytoplankton through *rbc*L genes and morphologically identification showed variation which was likely impacted by coastal freshwater input, winter monsoons, and upwelling. This study serves as the first molecular investigation of phytoplankton communities in the BOB. Inconsistent results were obtained via *rbc*L gene characterization vs. morphological identification suggesting that a careful coupling of molecular analysis and microscopic identification will be needed before the importance of these chromophytic phytoplankton, and their global distribution, are adequately understood.

## Author Contributions

LP designed the research, wrote the manuscript, and carried out the molecular and statistical analysis. CW carried out the sample collection and experimental analysis. NL, XS, MW, CZ, and GZ helped in the experimental analysis. JK drafted and proofread the manuscript. XW carried out the morphological identification of the phytoplankton. SJ designed the research and drafted the manuscript.

## Conflict of Interest Statement

The authors declare that the research was conducted in the absence of any commercial or financial relationships that could be construed as a potential conflict of interest.
