# Supplementary material for: Diversity and Spatial Distribution of Chromophytic Phytoplankton in the Bay of Bengal Revealed by RuBisCO Genes (rbcL)
Source: Front Microbiol. 2019 Jul 5;10:1501. doi: 10.3389/fmicb.2019.01501 (PMC6624743; doi:10.3389/fmicb.2019.01501)
Supplement: Supplementary file 1 [file Data_Sheet_1.docx]

**Table 1**: Quantification (cell L^-1^) and identification of phytoplankton communities based on microscopic morphology.

|  | | | | |  | |  | |  | |  | |  | |  | | **Stations** | |  | |  |  |  |  |
| --- | --- | --- | --- | --- | --- | --- | --- | --- | --- | --- | --- | --- | --- | --- | --- | --- | --- | --- | --- | --- | --- | --- | --- | --- |
| Species | | | | | BOB-1 | | BOB-2 | | BOB-3 | | BOB-4 | | BOB-5 | | BOB-6 | | BOB-7 | | BOB-8 | | BOB-9 | BOB-10 | BOB-11 | BOB-12 |
| **Bacillariophyta** | | | | |  | |  | |  | |  | |  | |  | |  | |  | |  |  |  |  |
| *Asterolampra marylandica* Ehrenberg | | | | | 2 | | 2 | | 0 | | 0 | | 0 | | 0 | | 0 | | 0 | | 0 | 0 | 0 | 0 |
| *Chaetoceros coarctatus* | | | | | 0 | | 0 | | 0 | | 0 | | 26 | | 0 | | 0 | | 0 | | 0 | 20 | 0 | 0 |
| *Chaetoceros decipiens f.decipiens* | | | | | 0 | | 0 | | 0 | | 0 | | 0 | | 0 | | 0 | | 0 | | 0 | 6 | 0 | 0 |
| *Chaetoceros curvisetus* | | | | | 0 | | 0 | | 0 | | 0 | | 0 | | 0 | | 0 | | 0 | | 0 | 0 | 16 | 0 |
| *Coscinodiscus granii* Grough | | | | | 0 | | 0 | | 0 | | 0 | | 2 | | 0 | | 0 | | 2 | | 0 | 0 | 2 | 0 |
| *Coscinodiscus radiatus* Ehrenberg | | | | | 0 | | 0 | | 0 | | 0 | | 0 | | 0 | | 8 | | 0 | | 0 | 0 | 0 | 0 |
| *Coscinodiscus subtilis* | | | | | 12 | | 28 | | 10 | | 0 | | 2 | | 8 | | 8 | | 4 | | 6 | 0 | 6 | 28 |
| *Coscinodiscus centralis* | | | | | 0 | | 0 | | 0 | | 2 | | 2 | | 0 | | 0 | | 0 | | 0 | 0 | 0 | 0 |
| *Eunotogramma debile* | | | | | 4 | | 2 | | 0 | | 0 | | 2 | | 196 | | 0 | | 4 | | 20 | 0 | 0 | 0 |
| *Fragilariopsis doliolus Medlin* P.A.Sims | | | | | 0 | | 0 | | 0 | | 20 | | 0 | | 0 | | 0 | | 0 | | 0 | 0 | 0 | 0 |
| *Hemidiscus cuneiformis* Wallich | | | | | 0 | | 0 | | 0 | | 0 | | 0 | | 0 | | 0 | | 0 | | 0 | 0 | 2 | 0 |
| *Hemiaulus sinensis* Greville | | | | | 0 | | 0 | | 0 | | 0 | | 0 | | 0 | | 10 | | 0 | | 0 | 0 | 10 | 0 |
| *Navicula spp.* | | | | | 0 | | 0 | | 0 | | 2 | | 4 | | 0 | | 0 | | 2 | | 0 | 0 | 0 | 0 |
| *Odontella sinensis* | | | | | 0 | | 0 | | 2 | | 0 | | 0 | | 0 | | 0 | | 0 | | 0 | 0 | 0 | 0 |
| *Planktoniella foromsa* | | | | | 0 | | 0 | | 0 | | 0 | | 0 | | 0 | | 4 | | 0 | | 0 | 0 | 0 | 0 |
| *Pleurosigma pelagicum* | | | | | 0 | | 0 | | 0 | | 0 | | 0 | | 2 | | 0 | | 0 | | 0 | 0 | 0 | 0 |
| *Rhizosolenia robusta* | | | | | 0 | | 0 | | 0 | | 0 | | 0 | | 0 | | 4 | | 0 | | 0 | 42 | 66 | 0 |
| *Rhizosolenia bergonii* Perty | | | | | 0 | | 0 | | 20 | | 0 | | 0 | | 0 | | 0 | | 0 | | 0 | 18 | 12 | 0 |
| *Synedra spp.* | | | | | 0 | | 0 | | 2 | | 4 | | 12 | | 0 | | 4 | | 2 | | 0 | 12 | 2 | 0 |
| *Thalassiothrix longissima* Cleve et Grunow | | | | | 0 | | 12 | | 0 | | 2 | | 10 | | 0 | | 0 | | 0 | | 0 | 2 | 0 | 0 |
| *Thalassionema frauenfeldii (Grunow)* Hallegraeff | | | | | 0 | | 0 | | 4 | | 0 | | 0 | | 0 | | 70 | | 0 | | 0 | 134 | 366 | 0 |
| Thalassiosira nordenskioldii | | | | | 0 | | 4 | | 0 | | 0 | | 0 | | 0 | | 0 | | 0 | | 0 | 0 | 2 | 0 |
| Bacillariophyta cells L^-1^ | | | | | 1.8×10^4^ | | 4.8×10^4^ | | 3.8×10^4^ | | 3×10^4^ | | 6×10^4^ | | 2.06×10^5^ | | 1.08×10^5^ | | 1.4×10^4^ | | 2.6×10^4^ | 2.34×10^5^ | 4.84×10^5^ | 2.8×10^4^ |
|  | | | | |  | |  | |  | |  | |  | |  | |  | |  | |  |  |  |  |
|  |  |  |  |  | |  | |  | |  | |  | |  | |  | |  | |  |  |  |  |  |
|  | | | | |  | |  | |  | |  | |  | |  | |  | |  | |  |  |  |  |
| **Dinoflagellates** | | | | |  | |  | |  | |  | |  | |  | |  | |  | |  |  |  |  |
| *Amphisolenia bidentata* | | | | | 0 | | 0 | | 0 | | 0 | | 2 | | 0 | | 0 | | 0 | | 2 | 0 | 0 | 0 |
| *Ceratium breve* var.parallelum | | | | | 0 | | 0 | | 0 | | 0 | | 0 | | 0 | | 0 | | 0 | | 0 | 4 | 2 | 0 |
| *Ceratium breve* var.breve | | | | | 0 | | 0 | | 0 | | 0 | | 0 | | 0 | | 0 | | 0 | | 0 | 0 | 2 | 0 |
| *Ceratium tripos* var.pulcbellum | | | | | 0 | | 0 | | 0 | | 0 | | 0 | | 0 | | 0 | | 0 | | 4 | 0 | 0 | 0 |
| *Ceratium bumile* | | | | | 0 | | 0 | | 0 | | 0 | | 0 | | 0 | | 0 | | 0 | | 0 | 0 | 0 | 2 |
| *Ceratium tripos var.atlanticum* | | | | | 0 | | 0 | | 0 | | 0 | | 0 | | 0 | | 4 | | 0 | | 0 | 0 | 0 | 16 |
| *Ceratium borridum var.denticulatum* | | | | | 0 | | 0 | | 0 | | 0 | | 2 | | 0 | | 6 | | 0 | | 0 | 0 | 0 | 0 |
| *Ceratium contrarium* | | | | | 0 | | 0 | | 0 | | 2 | | 0 | | 4 | | 8 | | 6 | | 6 | 12 | 6 | 6 |
| *Ceratium candelabrum* | | | | | 0 | | 0 | | 0 | | 0 | | 0 | | 0 | | 0 | | 0 | | 0 | 0 | 0 | 12 |
| *Ceratium deflexum* | | | | | 0 | | 0 | | 0 | | 0 | | 0 | | 0 | | 4 | | 0 | | 0 | 0 | 2 | 2 |
| *Ceratium digitatum* var.angusticornum | | | | | 0 | | 0 | | 0 | | 0 | | 0 | | 2 | | 0 | | 0 | | 0 | 0 | 0 | 0 |
| *Ceratium tripos* var.indicum | | | | | 0 | | 0 | | 0 | | 0 | | 0 | | 0 | | 0 | | 0 | | 8 | 0 | 0 | 0 |
| *Ceratium azoricum* | | | | | 0 | | 0 | | 0 | | 0 | | 0 | | 0 | | 0 | | 0 | | 0 | 0 | 2 | 0 |
| *Ceratium furca* | | | | | 2 | | 0 | | 0 | | 0 | | 2 | | 2 | | 0 | | 0 | | 12 | 4 | 4 | 54 |
| *Ceratium candelabrum* var.candelabrum | | | | | 0 | | 0 | | 0 | | 0 | | 0 | | 0 | | 0 | | 0 | | 4 | 0 | 0 | 0 |
| *Ceratium fusus var.seta* | | | | | 0 | | 0 | | 2 | | 2 | | 2 | | 4 | | 6 | | 0 | | 10 | 4 | 4 | 4 |
| *Ceratium macroceros* var.macroceros | | | | | 0 | | 0 | | 0 | | 0 | | 0 | | 0 | | 0 | | 0 | | 6 | 0 | 0 | 0 |
| *Ceratium massiliense* var.massiliense | | | | | 2 | | 0 | | 0 | | 0 | | 0 | | 0 | | 0 | | 0 | | 0 | 0 | 0 | 0 |
| *Ceratium massiliense* | | | | | 0 | | 0 | | 0 | | 0 | | 0 | | 0 | | 0 | | 0 | | 0 | 0 | 8 | 0 |
| *Ceratium tricboceros* | | | | | 0 | | 0 | | 0 | | 0 | | 2 | | 0 | | 0 | | 0 | | 0 | 0 | 0 | 0 |
| *Ceratocorys horrida* Stein 1 | | | | | 0 | | 4 | | 4 | | 8 | | 2 | | 4 | | 8 | | 0 | | 10 | 4 | 0 | 6 |
| *Dinophysis lativelata* | | | | | 0 | | 0 | | 0 | | 0 | | 0 | | 0 | | 0 | | 0 | | 0 | 2 | 0 | 0 |
| *Dinophysis apicata* | | | | | 0 | | 0 | | 0 | | 0 | | 0 | | 0 | | 0 | | 0 | | 2 | 0 | 0 | 0 |
| *Dinophysis laevis* | | | | | 2 | | 0 | | 0 | | 0 | | 0 | | 0 | | 0 | | 0 | | 0 | 0 | 0 | 0 |
| *Dinophysis caudata* | | | | | 0 | | 0 | | 0 | | 0 | | 0 | | 0 | | 0 | | 0 | | 0 | 0 | 2 | 0 |
| *Dinophysis doryphorum* | | | | | 0 | | 0 | | 2 | | 0 | | 0 | | 0 | | 0 | | 0 | | 0 | 0 | 0 | 0 |
| *Dinophysis tailisuni* | | | | | 0 | | 0 | | 0 | | 0 | | 0 | | 0 | | 0 | | 2 | | 0 | 0 | 0 | 0 |
| *Dinophysis acutoides* | | | | | 0 | | 0 | | 0 | | 0 | | 0 | | 2 | | 0 | | 0 | | 0 | 0 | 0 | 0 |
| *Dissodinium elegans* | | | | | 0 | | 0 | | 0 | | 0 | | 0 | | 0 | | 0 | | 0 | | 0 | 6 | 0 | 0 |
| *Gonyaulax polyedra* | | | | | 0 | | 0 | | 0 | | 0 | | 0 | | 0 | | 2 | | 0 | | 0 | 0 | 0 | 0 |
| *Gonyaulax minuta* | | | | | 2 | | 0 | | 2 | | 0 | | 0 | | 0 | | 0 | | 0 | | 0 | 0 | 0 | 0 |
| *Gonyaulax polygramma* | | | | | 0 | | 2 | | 2 | | 4 | | 0 | | 0 | | 0 | | 6 | | 0 | 0 | 0 | 0 |
| *Gymnodinium splendens* | | | | | 0 | | 0 | | 0 | | 4 | | 0 | | 0 | | 0 | | 0 | | 0 | 0 | 0 | 0 |
| *Gymnodinium vestifici* | | | | | 0 | | 0 | | 0 | | 0 | | 0 | | 0 | | 0 | | 0 | | 0 | 0 | 8 | 0 |
| *Gymnodinium viridescens* | | | | | 0 | | 6 | | 10 | | 0 | | 0 | | 2 | | 6 | | 4 | | 2 | 14 | 0 | 2 |
| *Histioneis cymbalaria* | | | | | 0 | | 0 | | 0 | | 0 | | 0 | | 0 | | 2 | | 0 | | 0 | 0 | 0 | 0 |
| *Histioneis elongata* | | | | | 0 | | 0 | | 0 | | 0 | | 0 | | 0 | | 0 | | 2 | | 0 | 0 | 0 | 0 |
| *Ornithocercus skogsbergii* | | | | | 0 | | 4 | | 0 | | 0 | | 0 | | 0 | | 0 | | 0 | | 0 | 0 | 0 | 0 |
| *Ornithocercus magnificus* Stein | | | | | 0 | | 0 | | 0 | | 2 | | 0 | | 0 | | 8 | | 0 | | 4 | 0 | 0 | 4 |
| *Ornithocercus thumii* | | | | | 0 | | 0 | | 0 | | 0 | | 0 | | 0 | | 0 | | 0 | | 2 | 0 | 0 | 0 |
| *Ornithocercus steinii* | | | | | 0 | | 4 | | 0 | | 0 | | 0 | | 0 | | 0 | | 0 | | 0 | 0 | 0 | 0 |
| *Oxytoxum scolopax* | | | | | 0 | | 2 | | 0 | | 0 | | 6 | | 0 | | 0 | | 0 | | 0 | 0 | 0 | 0 |
| *Oxytoxum globosum* | | | | | 0 | | 0 | | 0 | | 0 | | 0 | | 0 | | 0 | | 2 | | 0 | 0 | 0 | 0 |
| *Oxytoxum reticulatum* | | | | | 0 | | 0 | | 0 | | 0 | | 6 | | 0 | | 0 | | 0 | | 0 | 0 | 0 | 0 |
| *Pyropbacus borologium* | | | | | 0 | | 2 | | 0 | | 0 | | 0 | | 0 | | 0 | | 0 | | 2 | 0 | 0 | 0 |
| *Podolampas bipes* var.bipes | | | | | 0 | | 0 | | 0 | | 0 | | 2 | | 0 | | 2 | | 0 | | 6 | 0 | 0 | 0 |
| *Prorocentrum compressum* | | | | | 4 | | 22 | | 4 | | 10 | | 16 | | 26 | | 12 | | 4 | | 24 | 0 | 0 | 12 |
| *Prorocentrum dentatum* Stein | | | | | 0 | | 0 | | 0 | | 0 | | 0 | | 0 | | 0 | | 0 | | 0 | 0 | 0 | 0 |
| *Prorocentrum gracile* | | | | | 0 | | 0 | | 2 | | 6 | | 0 | | 16 | | 0 | | 0 | | 0 | 2 | 0 | 2 |
| *Prorocentrum leniculatum* | | | | | 0 | | 0 | | 2 | | 4 | | 6 | | 2 | | 6 | | 0 | | 0 | 0 | 0 | 0 |
| *Prorocentrum micans* Ehrenberg | | | | | 0 | | 4 | | 0 | | 10 | | 2 | | 20 | | 4 | | 0 | | 0 | 0 | 0 | 16 |
| *Protoperidinium biconicum* | | | | | 0 | | 0 | | 0 | | 0 | | 0 | | 0 | | 0 | | 0 | | 0 | 2 | 0 | 0 |
| *Protoperidinium latissimum* | | | | | 0 | | 0 | | 2 | | 0 | | 0 | | 0 | | 0 | | 0 | | 0 | 0 | 0 | 0 |
| *Protoperidinium curtipes* | | | | | 0 | | 2 | | 0 | | 0 | | 0 | | 0 | | 0 | | 0 | | 0 | 0 | 0 | 0 |
| *Protoperidinium grande* | | | | | 0 | | 0 | | 0 | | 0 | | 0 | | 0 | | 0 | | 0 | | 0 | 0 | 2 | 0 |
| *Protoperidinium inclinatum* | | | | | 0 | | 0 | | 0 | | 8 | | 0 | | 0 | | 0 | | 0 | | 0 | 0 | 0 | 0 |
| *Heteraulacus polyedricus* | | | | | 2 | | 0 | | 0 | | 0 | | 0 | | 0 | | 0 | | 4 | | 0 | 0 | 0 | 0 |
| *Protoperidinium tubum* | | | | | 0 | | 0 | | 0 | | 6 | | 0 | | 0 | | 8 | | 0 | | 0 | 2 | 0 | 0 |
| *Protoperidinium acbromaticum* | | | | | 0 | | 0 | | 0 | | 0 | | 0 | | 0 | | 0 | | 0 | | 0 | 0 | 0 | 2 |
| *Protoperidinium longicollum* | | | | | 0 | | 0 | | 0 | | 0 | | 0 | | 0 | | 0 | | 0 | | 2 | 0 | 0 | 0 |
| *Protoperidinium subinerme* | | | | | 2 | | 2 | | 0 | | 4 | | 0 | | 0 | | 2 | | 0 | | 2 | 0 | 0 | 2 |
| *Protoperidinium biconicum* | | | | | 0 | | 0 | | 0 | | 2 | | 0 | | 0 | | 0 | | 0 | | 0 | 0 | 0 | 0 |
| *Protoperidinium subpyriforme* | | | | | 2 | | 2 | | 0 | | 0 | | 0 | | 0 | | 0 | | 0 | | 0 | 2 | 0 | 0 |
| *Protoperidinium sourniai* | | | | | 0 | | 0 | | 0 | | 0 | | 0 | | 2 | | 0 | | 0 | | 0 | 0 | 0 | 0 |
| *Pyrocystis fusiformis* | | | | | 0 | | 0 | | 0 | | 2 | | 0 | | 0 | | 0 | | 0 | | 0 | 0 | 0 | 0 |
| *Pyrocystis robusta* | | | | | 0 | | 0 | | 2 | | 12 | | 8 | | 0 | | 2 | | 0 | | 2 | 0 | 0 | 0 |
| *Pyrocystis noctiluca* | | | | | 0 | | 2 | | 0 | | 2 | | 0 | | 2 | | 0 | | 0 | | 0 | 0 | 0 | 0 |
| *Dissodinium gerbaultii* | | | | | 0 | | 0 | | 4 | | 0 | | 0 | | 0 | | 0 | | 0 | | 0 | 0 | 0 | 0 |
| *Pyropbacus vancampoae* | | | | | 0 | | 0 | | 0 | | 0 | | 0 | | 0 | | 0 | | 0 | | 0 | 0 | 2 | 0 |
| *Scrippsiella trochoidea* (Stein) Loeblich | | | | | 0 | | 0 | | 0 | | 0 | | 0 | | 0 | | 0 | | 2 | | 0 | 0 | 0 | 0 |
| *Heteraulacus polyedricus* | | | | |  | |  | |  | |  | |  | |  | | 4 | |  | |  |  |  |  |
| Dinoflagellates cells L^-1^ | | | | | 1.8×10^4^ | | 5.8×10^4^ | | 3.8×10^4^ | | 8.8×10^4^ | | 5.8×10^4^ | | 8.8×10^4^ | | 9.4×10^4^ | | 3.2×10^4^ | | 1.1×10^5^ | 5.8×10^4^ | 4.4×10^4^ | 1.42×10^5^ |
|  | | | | |  | |  | |  | |  | |  | |  | |  | |  | |  |  |  |  |
| **Chrysophyta** | | | | |  | |  | |  | |  | |  | |  | |  | |  | |  |  |  |  |
| *Dictyocha fibula* | | | | | 0 | | 0 | | 0 | | 16 | | 0 | | 0 | | 8 | | 8 | | 0 | 0 | 0 | 8 |
| Chrysophyta cells L^-1^ | | | | | 0 | | 0 | | 0 | | 1.6×10^4^ | | 0 | | 0 | | 8×10^3^ | | 8×10^3^ | | 0 | 0 | 0 | 8×10^3^ |
|  | | | | |  | |  | |  | |  | |  | |  | |  | |  | |  |  |  |  |
| **Cyanophyta** | | | | |  | |  | |  | |  | |  | |  | |  | |  | |  |  |  |  |
| *Trichodesmium thiebaultii* Gomont | | | | | 0 | | 0 | | 0 | | 0 | | 140 | | 0 | | 0 | | 0 | | 5060 | 0 | 2000 | 0 |
| *Richelia intracellularis* | | | | | 0 | | 0 | | 120 | | 0 | | 40 | | 96 | | 0 | | 0 | | 0 | 0 | 0 | 0 |
| Cyanobacteria cells L^-1^ | | | | | 0 | | 0 | | 1.2×10^5^ | | 0 | | 1.8×10^5^ | | 9.6×10^4^ | | 0 | | 0 | | 5.6×10^6^ | 0 | 2×10^6^ | 0 |


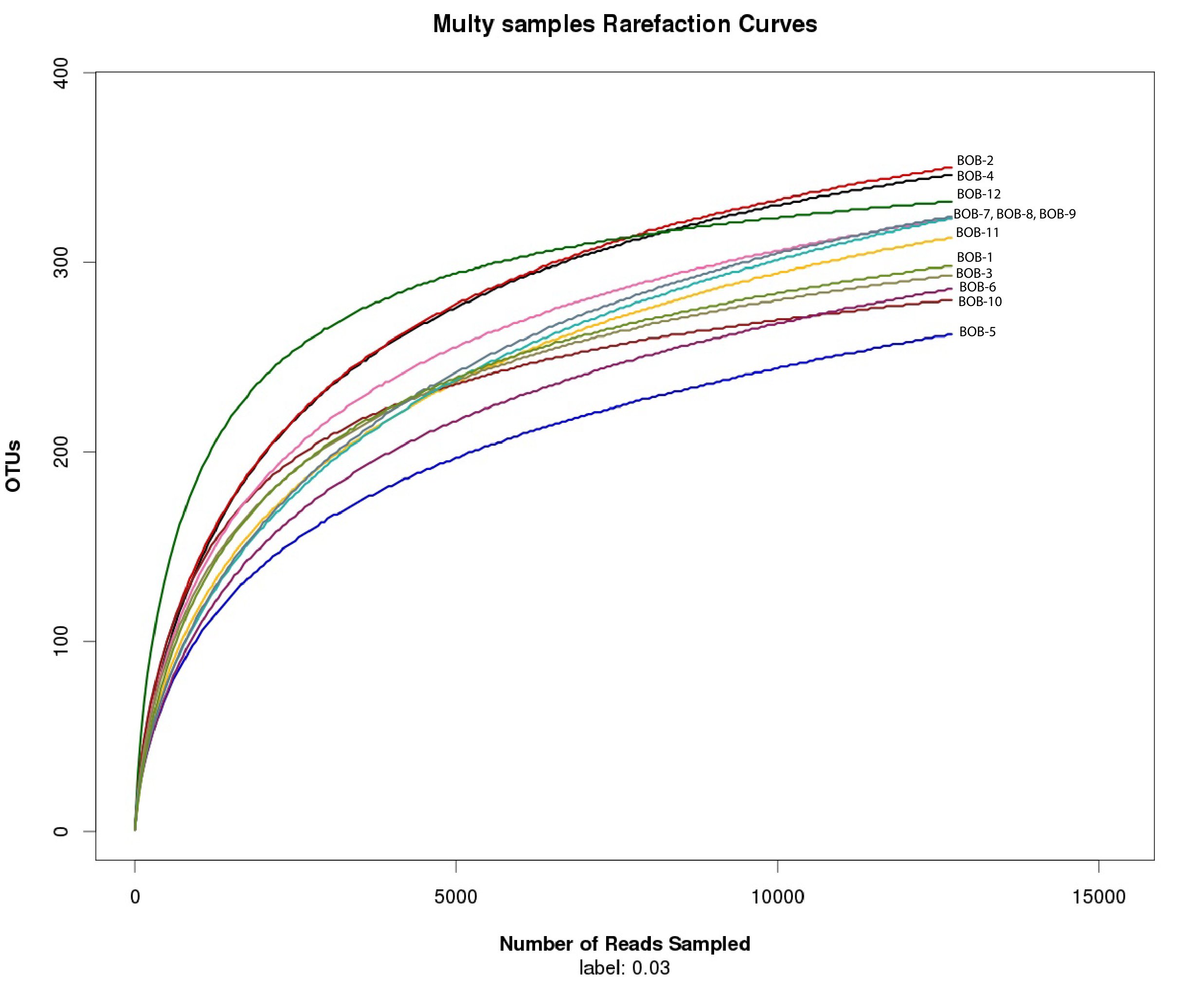


**Figure 1.** Rarefaction curves comparing the number of reads to the number of phylotypes (OTUs) found in the BOB samples
